# Geochemical Contamination, Speciation, and Bioaccessibility of Trace Metals in Road Dust of a Megacity (Guangzhou) in Southern China: Implications for Human Health

**DOI:** 10.3390/ijerph192315942

**Published:** 2022-11-29

**Authors:** Fei Tang, Zhi Li, Yanping Zhao, Jia Sun, Jianteng Sun, Zhenghui Liu, Tangfu Xiao, Jinli Cui

**Affiliations:** 1 Key Laboratory for Water Quality and Conservation of the Pearl River Delta, Ministry of Education, School of Environmental Science and Engineering, Guangzhou University, Guangzhou 510006, China; 2 Guangdong Provincial Key Laboratory of Petrochemical Pollution Processes and Control, School of Environmental Science and Engineering, Guangdong University of Petrochemical Technology, Maoming 525000, China; 3 Guangdong Provincial Key Laboratory of Chemical Measurement and Emergency Test Technology, Institute of Analysis, China National Analytical Center, Guangdong Academy of Sciences, Guangzhou 510070, China

**Keywords:** bioaccessibility, chemical sequential extraction, health risk assessment, street dust, trace metal contamination

## Abstract

Road dust has been severely contaminated by trace metals and has become a major health risk to urban residents. However, there is a lack of information on bioaccessible trace metals in road dust, which is necessary for an accurate health risk assessment. In this study, we collected road dust samples from industrial areas, traffic intersections, and agricultural fields from a megacity (Guangzhou), China, and conducted a geochemical enrichment, speciation, and bioaccessibility-based health risk assessment of trace metals. In comparison with local soil background values, the results revealed a significant accumulation of trace metals, including Zn, Cd, Cu, and Pb in the road dust, which is considered moderate to heavy pollution. Sequential extraction indicated that most trace metals in the road dust were primarily composed of a Fe/Mn oxide-bound fraction, carbonate-bound fraction, and residual fraction, while the dominant fraction was the organic matter-bound fraction of Cu, and the residual fractions of As, Cr, and Ni. The in vitro gastrointestinal (IVG) method revealed that high percentages of Zn, Cd, Cu, and As were bioaccessible, suggesting the possible dissolution of trace metals from adsorbed and carbonate-associated fractions in road dust exposed to the biological fluid matrix. The IVG bioaccessibility-based concentration largely decreased the noncarcinogenic health risk to a negligible level. Nevertheless, the entire population is still exposed to the cumulative probability of a carcinogenic risk, which is primarily contributed to by As, Cd, Cr, and Pb. Future identification of the exact sources of these toxic metals would be helpful for the appropriate management of urban road dust contamination.

## 1. Introduction

During the rapid global urbanization process, various anthropogenic activities have released a large number of trace metals into the urban environment [1]. Severe contamination by trace metals, including Pb, Zn, As, Cr, and Ni, in urban soil and street dust [2,3,4] is harmful to local residents, particularly children, via direct ingestion or inhalation [5,6]. Due to limited urban land resources, comprehensive contamination condition surveys and health risk assessments of urban environments are required for safe land reutilization.

Road dust, which includes various anthropogenic-induced trace metals, is a key factor that impacts human health in urban cities [7,8]. The main sources of trace metals in complex road dust include natural sources such as the soil weathering matrix [9,10], industrial activities [2], agricultural production [11], and vehicle emissions [12,13]. Observed geochemical hotspots of trace metals are often contributed to by dense industrial and traffic activity [14,15,16]. The anthropogenic activity-associated Pb, As, Cr, and Ni in road dust are usually major metals that have the greatest impact on noncancer or cancer risks to humans [17,18]. However, the use of total concentrations to calculate the uptake of trace metals from road dust into the human body overestimates the potential health risk because some immobile fractions are not bioaccessible [19].

A bioaccessibility-corrected assessment is necessary to estimate the potential risk of road dust to human health. However, in comparison with urban soil, information on bioaccessible or extractable trace metal fractions in urban dust is limited [11]. Recently, a few studies have indicated a significant decrease in the health risks of trace metals in street dust because only some of them are bioaccessible, such as 50% of Pb and Zn from Kolkata and Bengaluru in India [20], 15.0–43.7% of trace metals from Chengdu in China [21], and 43 ± 9% of Pb across five northern UK cities [22]. Higher bioaccessibility of trace metals was observed in dust from commercial and traffic areas than in residential and park areas of Chengdu [21]. The large variation in the bioaccessible fractions of different trace metals in road dust environments may be dependent on their chemical speciation and origination [10,23]. This origination- or speciation-related bioaccessibility information is critical for accurately assessing ecological and health risks and for developing appropriate control strategies for urban contamination [24,25,26]. However, related information is largely unavailable.

The megacity of Guangzhou is a region in the Pearl River Delta that has developed rapidly over the last several decades. Previous studies have confirmed that road dust and surface soil in Guangzhou were severely contaminated by trace metals, such as Cu, Zn, Pb, and Ni [5,8,16,27]. Therefore, we aimed to evaluate the trace metal geochemistry and ecological and health risks of road dust from agricultural, industrial, and traffic intersection areas in Guangzhou, by integrating chemical species and bioaccessibility analysis. This study provides crucial information on the accurate assessment of urban environmental contamination and could be helpful for global land reutilization.

## 2. Materials and Methods

### 2.1. Study Site, Sampling, and Analysis

Guangzhou (113°7′37″~113°31′21″ N, 22°44′2″~22°23′51″ E) is located in the center of the Pearl River Delta in southern China. It has warm and humid weather with an average precipitation of 1600–2300 mm/year and an annual temperature variation of approximately 15 °C [28]. Guangzhou is one of the most developed megacities in China with a residential population reaching approximately 18,900,000 and rapid industrialization over the last three decades, contributing to 2.47% of the total GDP of China [29,30]. Currently, Guangzhou is an international trade center and comprehensive transportation hub.

From December 2020 to January 2021, road dust particles were collected in the absence of precipitation for more than seven days from key industrial parks (*n* = 23), main traffic roads (*n* = 20), and typical agricultural fields (*n* = 5) using a dustpan and brush [16]. The samples were placed in polyethylene bags, transferred to the lab, and stored in the dark before sample preparation.

The road dust samples were air-dried, sieved through a 2 mm polyethylene sieve to remove stones, coarse materials, and other debris, and ground into powder before analysis. Trace metals in road dust samples were subjected to pseudo-total digestion using HNO_3_, HCl, HF, and HClO_4_ [31] and analyzed using MP-AES (4200, Agilent, Australia) or ICP-MS (7700, Agilent, CA, USA). Quality assurance and quality control (QA/QC) were performed using a standard reference material (GBW07405), reagent blank, and triplicate analysis. The recovery rates of all the elements in these standard materials remained within 83.5–123.5%. Triplicate samples indicated a bias ranging within 9.2% for all the metals; average values of trace metals were reported.

### 2.2. Trace Metal Contamination Assessment

The geological accumulation index (I_geo_) (Table S1) was employed to estimate the contamination degree of trace metals in road dust [32]:

I_geo_ = log_2_(C_i_/1.5B_i_) (1)

where I_geo_ is unitless, C_i_ is the elemental concentration in road dust, B_i_ is the geochemical background concentration in Guangzhou, and the coefficient 1.5 is generally used to adjust the natural lithologic variations in the background values [32].

Because I_geo_ can only assess the pollution of individual elements, the improved Nemerow index (INI) was used to assess the ecological risk of all the analyzed trace metals [33]:

(2) $$\mathrm{INI}=\sqrt{\frac{{\mathrm{Igeo}}_{\max}^{2}+{\mathrm{Igeo}}_{\mathrm{avg}}^{2}}{2}}.$$

where I_geomax_ and I_geoavg_ are the maximum value and average value of I_geo_ for trace metals, respectively. The classification levels of INI are shown in Supplementary Material Table S2.

The potential ecological risk index (RI) [34] was used to evaluate the degree of environmental risk caused by the analyzed trace metals in road dust:

(3) $$\mathrm{RI}=\sum_{i=1}^{n}E_{r}^{i}=\sum_{i=1}^{n}T_{r}^{i}C_{f}^{i}=\sum_{i=1}^{n}T_{r}^{i}C_{D}^{i}/C_{b}^{i}.$$

where RI (no unit) is the total potential ecological risk index, $E_{r}^{i}$ (no unit) is the single ecological risk index for a given metal element, the $T_{r}^{i}$ value is the toxicity response coefficient of trace metal i (no unit), and the $T_{r}^{i}$ values were Cu (5), Cr (2), Ni (5), Zn (1), Pb (5), Cd (30), As (10), and V (2) [34]. $C_{f}^{i}$ (no unit) is the contamination coefficient element calculated using the analyzed concentration ($C_{D}^{i}$) and background value ($C_{b}^{i}$) of trace metal i. The RI classifications of trace metal pollution are in the order of low ecological risk (RI ≤ 150), moderated ecological risk (150 ≤ RI < 300), considerable ecological risk (300 ≤ RI < 600), and high ecological risk (RI ≥ 600) [34].

Spatial variation characteristics of trace metals in road dust were explored by ArcGIS 10.6 [16]. Inverse distance weighting interpolation (IDW) from the Spatial Analyst toolbox was selected during the spatial distribution analysis of the total concentrations, the improved Nemerow index, and the ecological risk index.

### 2.3. Sequential Fractions and Bioaccessibility Assessment of Trace Metals in Road Dust

Five operational sequential fractions were investigated according to our previous study [35], and as follows: F1, exchangeable fraction; F2, carbonate-bound and specifically adsorbed fraction (acid-extractable part); F3, Fe/Mn oxide fraction (reducible part); F4, organic/sulfide fraction (oxidizable part); F5, residual fraction, which is subtracted from the corresponding pseudo-total digested result.

A typical bioaccessibility test of the in vitro gastrointestinal (IVG) method employing both gastric and intestinal phases was employed to investigate the potential mobilizable fractions of trace metals in the road dust once ingested [36,37]. Briefly, one gram of road dust samples was accurately weighed and added to 150 mL of gastric phase fluid (10 g pepsin and 8.77 g NaCl in one liter of deionized water, pH 2.5) and agitated horizontally at 150 rpm at 37 °C. The suspension solutions were then centrifuged at 4000 rpm for 10 min, and 10% of the gastric phase supernatant was sampled. To the remainder of the gastric phase solutions were added an appropriate amount of bile (3.5 g/L) and pancreatin (0.35 g/L) and the total solution was adjusted to pH 5.5 with Na_2_CO_3_, which is considered as intestinal phase extraction. All of the solution samples were centrifuged, filtered through a 0.22 μm filter, and digested prior to analysis. The bioaccessibility (BA%) of trace metals was calculated as percentages of the bioaccessible fractions, including gastric and intestinal phases, in the total content of dust samples.

### 2.4. Human Health Risk Assessment and Monte Carlo Simulation

Health risk assessments of trace metals are mainly based on models developed by the US Environmental Protection Agency [38,39]. Rather than total concentrations, IVG-based bioaccessible contents of trace metals were used to estimate the possible uptake quantity in the human body. Three pathways for the possible introduction of road dust into the human body, namely, bioaccessibility-adjusted ingestion (ADD_ing_), inhalation (ADD_inh_), and skin contact (ADD_dermal_) were considered when calculating the average daily absorbed dose (ADD) of trace metals following the three Equations (4)–(6):

(4) $${\mathrm{ADD}}_{\mathrm{ing}}=C\times \mathrm{BA}\times \frac{{\mathrm{Ing}}_{R}\times \;\mathrm{EF}\;\times \;\mathrm{ED}}{\mathrm{BW}\;\times \;\mathrm{AT}}\times 10^{-6}.$$

(5) $${\mathrm{ADD}}_{\mathrm{inh}}=C\times \frac{{\mathrm{Inh}}_{R}\times \;\mathrm{EF}\;\times \;\mathrm{ED}}{\mathrm{PEF}\;\times \;\mathrm{BW}\;\times \;\mathrm{AT}}.$$

(6) $${\mathrm{ADD}}_{\mathrm{dermal}}=C\times \frac{\mathrm{AF}\;\times \;\mathrm{SA}\;\times \;\mathrm{ABS}\;\times \;\mathrm{EF}\;\times \;\mathrm{ED}}{\mathrm{BW}\;\times \;\mathrm{AT}}\times 10^{-6}.$$

Here, C is the trace metal concentration; BA is the bioaccessibility; IngR and InhR are soil ingestion and inhalation rate, respectively; EF, ED, and BW are exposure frequency (day/year), exposure duration (year), and body weight (kg), respectively. In Equation (6), AF, SA, ABS, and AT are adherence factor (mg/m^2^/day), exposed skin area (cm^2^), dermal absorption factor, and average time (days), respectively. The parameter details can be found in previous studies [27,40,41] and are provided in the Supplementary Material Table S3.

For carcinogenic trace metals, the lifetime average daily absorbed doses (LADD_inh_) were used to assess the effects of the five carcinogenic trace metals (As, Pb, Ni, Cr, Cd) on adults and children using Equations (7)–(9):

(7) $${\mathrm{LADD}}_{\mathrm{ing}}=C\times \mathrm{BA}\times \frac{\mathrm{EF}}{\mathrm{AT}\times \mathrm{PEF}}\times \left(\frac{{\mathrm{IngR}}_{\mathrm{child}}\times {\mathrm{ED}}_{\mathrm{child}}}{{\mathrm{BW}}_{\mathrm{child}}}+\frac{{\mathrm{IngR}}_{\mathrm{adult}}\times {\mathrm{ED}}_{\mathrm{adult}}}{{\mathrm{BW}}_{\mathrm{adult}}}\right)\times 10^{-6}.$$

(8) $${\mathrm{LADD}}_{\mathrm{inh}}=C\times \frac{\mathrm{EF}}{\mathrm{AT}\times \mathrm{PEF}}\times \left(\frac{{\mathrm{InhR}}_{\mathrm{child}}\times {\mathrm{ED}}_{\mathrm{child}}}{{\mathrm{BW}}_{\mathrm{child}}}+\frac{{\mathrm{InhR}}_{\mathrm{adult}}\times {\mathrm{ED}}_{\mathrm{adult}}}{{\mathrm{BW}}_{\mathrm{adult}}}\right).$$

(9) $${\mathrm{LADD}}_{\mathrm{dermal}}=\frac{C\times \mathrm{EF}\times \mathrm{ABS}}{\mathrm{AT}}\times \left(\frac{{\mathrm{AF}}_{\mathrm{child}}\times {\mathrm{SA}}_{\mathrm{child}}\times {\mathrm{ED}}_{\mathrm{child}}}{\mathrm{BWchild}}+\frac{{\mathrm{AF}}_{\mathrm{adult}}\times {\mathrm{SA}}_{\mathrm{adult}}\times {\mathrm{ED}}_{\mathrm{adult}}}{{\mathrm{BW}}_{\mathrm{adult}}}\right)\times 10^{-6}.$$

Here, PEF is the particle emission factor; ABS is the dermal absorption factor (Table S3); the other parameters are the same as that in the ADD calculation [41,42,43].

The risk factor (HQ) was used to calculate the noncarcinogenic effects of trace metals in road dust employing a reference dose (RfD), and the hazard rate (HI) was equal to the sum of the risk factors for multiple pathways or substances [42]. The possible occurrence of noncarcinogenic effects was suggested using an HI value higher than one; higher HI values indicate a higher probability [27,31].

(10) $$\mathrm{HQ}=\frac{{\mathrm{ADD}}_{\mathrm{ing}/\mathrm{inh}/\mathrm{dermal}}}{\mathrm{RfD}}.$$

(11) HI=∑HQi.

The cancer coefficient (CR) was used to estimate a person’s lifetime risk of cancer using a slope factor (SF). The total cancer risk (TR) is the sum of the CR of the three routes [43].

The slope factor was used to assess the carcinogenic risk due to the lifetime exposure to trace metals. The carcinogenic risk and noncarcinogenic risk of each trace metal were calculated using the following equations. Values of CR lower than 10^−6^ (a probability of 1 in 1,000,000 of an individual developing cancer) were considered negligible (acceptable), while a CR value higher than 10^−4^ was considered unacceptable.

(12) $$\mathrm{CR}={\mathrm{LADD}}_{\mathrm{ing}}\times \mathrm{SF}$$

(13) TR=∑CRi.

Considering the point estimation and lack of actual values of various parameters in the USEPA’s human health risk model, a Monte Carlo simulation was further used in the study to provide specific guidance for risk control by estimating multimedia exposure and parameter sensitivity qualification [44]. The trace metal concentration, body weight, and inhalation rates were varied during modeling, and log-normal distributions of each variable were used. The Monte Carlo analysis was performed in Oracle Crystal Ball software for a total of 10,000 interactions to ensure convergence results.

## 3. Results

### 3.1. Contamination, Enrichment, and Ecological Risk of Trace Metals in Road Dust

The concentrations of the elements obtained from the collected samples are shown in Table 1. Except for V, all of the trace metals showed an average concentration higher than the background values in Guangzhou. High concentrations of trace metals were observed in road dust, reaching 2.4–13.5 times that from industrial parks, 2.3–12.0 times that from traffic roads, and 1.0–11.7 times that from agricultural fields. The four most enriched trace metals, including Cd, Zn, Cu, and Pb, were significantly observed, followed by Cr, Ni, As, and V.

In the three kinds of urban functional regions, a lack of significant difference was observed for the analyzed trace metals, except for Zn (*p* < 0.01). Zn was highest in industrial regions (640 ± 493 mg/kg), followed by traffic intersections (368 ± 166 mg/kg) and agricultural fields (143 ± 57.5 mg/kg).

**Figure 1:**
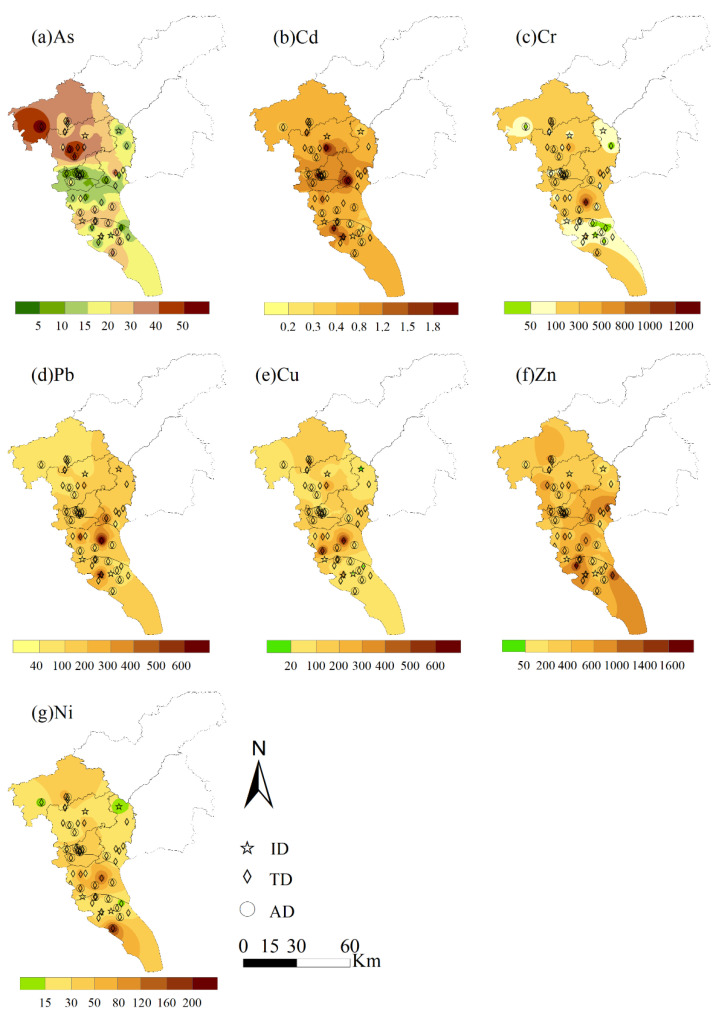
Spatial distribution of trace metal concentrations in road dust from the industrial park (ID), traffic intersection (TD), and agricultural field (AD) areas of Guangzhou.

A high heterogeneous distribution of trace metals was identified in road dust from Guangzhou (Figure 1). Several hotspot regions were observed for Cd, Pb, Cu, Cr, and Zn in Tianhe District and adjacent districts of Huangpu, Baiyun, and Haizhu. Arsenic and V showed much lower concentrations of 4.7–61.2 mg/kg and 18.3–61.8 mg/kg, respectively, exhibiting no obvious enrichment (Table 1). In contrast to other trace metals, a higher content of As was distributed in suburban areas (Figure 1a) without the observation of significant anthropogenic activities.

**Figure 2:**
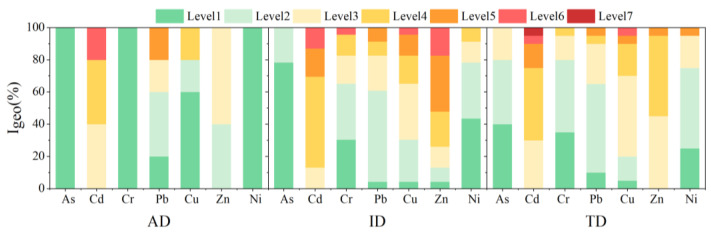
Geological accumulation index (I_geo_) of trace metals in the road dust from the industrial park (ID), traffic intersection (TD), and agricultural field (AD) areas of Guangzhou.

The geological accumulation index values (I_geo_) showed that road dust in Guangzhou was mainly moderately to heavily polluted (Figure 2). Road dust from industrial parks showed the highest concentration range compared to traffic intersections and agricultural fields. For the industrial park dust, the contamination level was highest for Zn (3.2), followed by Cd (3.0) and Cu (2.4); accordingly, 52%, 30%, and 17% of the samples were heavily contaminated by Zn, Cu, and Cu, respectively. At traffic intersections, Cd showed the highest concentration level with an order of magnitude of 7 in some areas, followed by Zn (2.4) and Cu (2.2). In the road dust from agricultural fields, most samples showed a moderate to heavy contamination of Cd. One agricultural road dust sample was extremely contaminated by Cd and Pb, near the industrial zone, revealing the contamination by industrial activities in the surrounding region.

**Figure 3:**
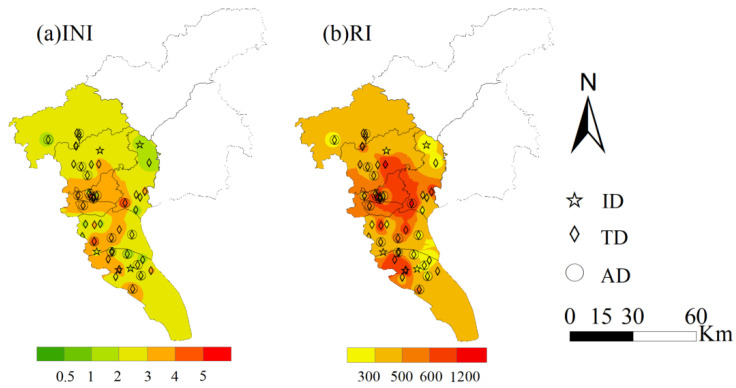
Spatial distribution for the improved Nemerow index (INI) and ecological risk index (RI) for the trace metals in the road dust from the industrial park (ID), traffic intersection (TD), and agricultural field (AD) areas of Guangzhou.

Based on I_geo_, the INI calculation estimated the ecological risk of all the analyzed trace metals and revealed that all the dust samples showed INI values higher than 1, with a mean value of 2.8 and a median value of 2.7 (Figure 3a). Most dust samples (73%) reached the level of “moderately to heavily contaminated” (INI ≥ 2) and above. One site reached the level of “heavily to extremely contaminated” (INI ≥ 5), and three sites reached the level of severe contamination, “Heavily to extremely contaminated” (4 ≤ INI < 5). Considering the INI for each kind of trace metal, all of the samples were under “considerable risk” from Cd, and 42% and 51% of the samples were at an “extremely high risk” and “high risk”, respectively (Figure 3b). A “moderate risk” was suggested from Zn (51%) and Cu (23%). Low risk was estimated in road dust from As, Ni, and Cr.

Similar to INI, the mean ecological risk value (RI) of urban dust trace metals in Guangzhou was 495 (159–1698), which is considered high ecological risk (Figure 3b). Considering the spatial distribution in different districts, the RI mean values showed a general sequence of Yuexiu (236–1698, mean of 794), Panyu (206–1021, mean of 474), Nansha (159–1219, mean of 459), Tianhe (325–550, mean of 453), and Huadu (218–595, mean of 416). Road dust showed the highest mean RI values in industrial regions (523 ± 312), followed by traffic intersections (477 ± 374) and agricultural regions (444 ± 438).

### 3.2. Sequential Chemical Species of Trace Metals in Road Dust

**Figure 4:**
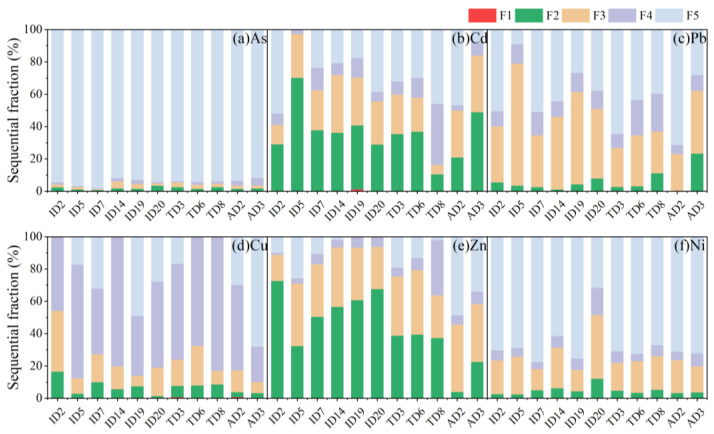
Chemical fractions of trace metals in the road dust from the industrial park area (ID), traffic intersection (TD), and agricultural field (AD) areas of Guangzhou using the sequential extraction procedure: exchangeable fraction (F1), carbonate-bound and specifically adsorbed fraction (acid extractable, F2), Fe/Mn oxide fraction (reducible, F3), organic/sulfide fraction (oxidizable, F4), and a residual fraction (F5). F5 was determined via subtraction from the pseudo-total digested result.

Sequential extraction experiments were explored to determine the chemical availability and possible mobilization of trace metals from road dust [2]. As shown in Figure 4, the residual fraction dominated for As (91.6–98.2%) and Ni (31.6–77.5%), while Pb and Cd were composed of both the residual fraction (5.5–23.4% for Pb, 0.1–51.8% for Cd) and the Fe/Mn oxide-bound fraction (23.1–75.5% for Pb, 5.1–35.8% for Cd). The carbonate-bound fraction (4.0–72.7%) and Fe/Mn oxide-bound fraction (16.1–41.7%) contributed to the main composition of Zn in road dust. A much higher percentage of Cu was found in the organic matter-bound fraction (22.0–82.9%).

In the three kinds of road dust, no significant difference could be observed in the sequential fractionation distribution of trace metals, particularly for industrial and traffic intersections. There was some difference in the agricultural road dust sample AD2, which showed much lower carbonate-bound Zn and higher residual Zn (Figure 4d). Similarly, AD2 exhibited higher residual Cd and Pb. The different distribution of trace metals in road dust may be associated with the physiochemical properties of the dust composite.

### 3.3. IVG Bioaccessibility Analysis of Trace Metals in Road Dust

**Figure 5:**
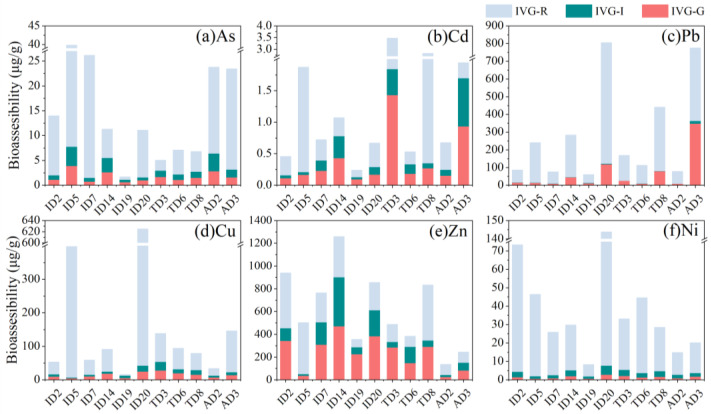
Bioaccessible trace metal concentrations in the simulated gastric (IVG-G) and intestinal (IVG-I) phases using the in vitro gastrointestinal (IVG) method for road dust from the industrial park (ID), traffic intersection (TD), and agricultural field (AD) areas of Guangzhou. IVG-R was determined via subtraction from the pseudo-total digested result.

Trace metals in dust particulates from urban environments often pose serious health risks to populations via incidental ingestion, especially for children, who typically ingest dust through the hand-to-mouth pathway [46]. The simulated IVG tests indicated that significantly higher contents of trace metals were bioaccessible in the gastric phases than in the intestinal phases (Figure 5). The IVG results showed that a much higher percentage of trace metals was bioaccessible, such as Zn (57.1 ± 21.7%), Cd (47.4 ± 23.3%), Cu (30.6 ±20.5%), Pb (17.4 ± 10.8%), As (16.6 ± 20.3), and Ni (13.1 ± 6.3). No significant difference was found in the distribution of trace metal bioaccessibility in different functional areas. The IVG bioaccessible As in road dust was much higher than the potential mobilizable fractions performed in the sequential extraction experiment.

### 3.4. IVG Bioaccessibility-Based Health Risk Assessment Using Monte Carlo Simulations

**Figure 6:**
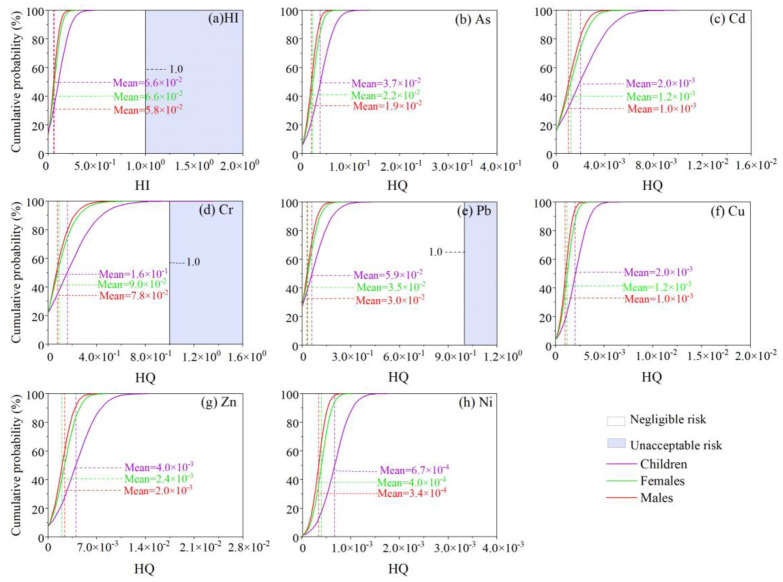
Hazard quotient (HQ) of the cumulative probability of noncarcinogenic risk of each trace metal and hazard index (HI) values for the cumulative probability of total noncarcinogenic risk in road dust from Guangzhou.

According to the human health risk model and Monte Carlo simulation, the cumulative probability of health risks from the trace metals in the road dust was estimated. Total trace metal concentrations were first used in the calculation of noncarcinogenic risk (HI), showing an order of children> adult women> adult men (Table 2 and Figure 6). All of the HI values for adults were below 1, revealing a negligible noncarcinogenic risk. However, the noncarcinogenic risk value for children reached 1.2 when the probability exceeded 95%. When bioaccessible contents were used, the noncarcinogenic risk for children largely decreased to 2.7 × 10^−1^ at a cumulative probability of 95% (Table 2). Although road dust showed no significant enrichment of Cr, it showed the highest contribution to the noncarcinogenic health risk (Figure 6).

**Figure 7:**
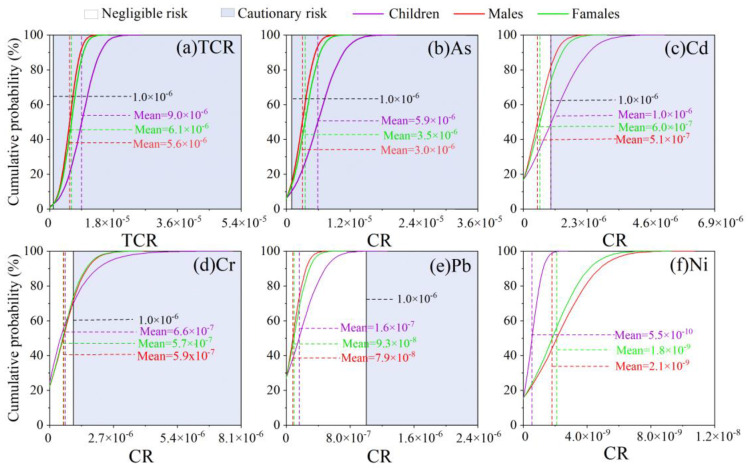
Cumulative probability of carcinogenic risk (CR) and cumulative probability of total carcinogenic risk (TCR) for trace metals in road dust from Guangzhou.

The cumulative probability of carcinogenic risk (CR) for each trace metal and total carcinogenic risk (TCR) for road dust is shown in Figure 7 and Table 3. In comparison with the acceptable threshold of 1.0 × 10^−6^, a much higher percentage of the population exceeded this value: 97.7% for children, 97.8% for adult females, and 97.4% for adult males. Furthermore, the mean value of the TCRs for the five trace metals were several times the threshold (Table 3), such as children (2.1–25.6 times) > adult females (1.7–15.0 times) > adult males (1.7–16.3 times). The results showed a cautionary carcinogenic risk for trace metals, especially As, Cd, Cr, and Pb, from most road dust samples to all the populations.

## 4. Discussion

The higher enrichment of Cd, Zn, Cu, and Pb than Cr, Ni, and As in road dust from Guangzhou is generally similar to a recent report from the Pearl River Delta [47]. The severe contamination of Cd, Pb, Cu, Cr, and Zn in the Tianhe District and the adjacent districts of Huangpu, Baiyun, and Haizhu is generally consistent with previous reports [5,16]. The spatial distribution analysis of trace metal concentrations, geochemical enrichment, and ecological risks indicated greater contamination conditions from the traditional central districts of Guangzhou. The higher INI and RI ecological risk observed in the central Guangzhou districts are probably due to traffic activities and industrially emitted residues [5,16,48]. The INI calculation indicated that Cd in road dust posed a major ecological risk in Guangzhou due to its high toxicity even at low concentrations [1,27].

Some sampling sites from the newly developing regions in the Huangpu, Panyu, and Nansha districts were also contaminated by trace metals. Road dust from some industrial areas in Nansha showed high concentrations of Cd, Cr, Ni, and Zn, confirming the occurrence of deteriorating point pollution from industrial emissions [14].

Anthropogenically sourced Pb, Zn, and Cd are commonly adsorbed by Fe/Mn oxide or precipitated as carbonate minerals in the environmental matrix [35]. The higher affinity of Cu for organic components in road dust resulted in higher percentages of the organic matter-bound fraction [23,49]. The distribution of sequential fractionation for these trace metals is consistent with other studies of road dust [2,23,50,51]. The carbonate-bound fraction, Fe/Mn oxide-bound fraction, and some clay mineral-adsorbed trace metals are probably dissociated or desorbed at changing pH levels or in the presence of organic acids or competitive ions [32,35,52].

The dominant species of As in road dust is the hardly mobilized residual fraction. The enrichment and spatial distribution analysis suggested that As probably originated from natural sources, which are usually immobile under mild environments [10]. High background As values of 0.07–197 mg/kg (11.6 ± 26.8 mg/kg) were observed in the soil in the nearby city of Shenzhen [53]. This is because the red soil of the Pearl River Delta is primarily developed from the weathering products of granite, sandy shale, and limestone, which may contain high concentrations of As [54,55].

Rather than the total concentration, only bioaccessible fractions of trace metals in road dust can be ingested [40] and were largely decreased for biological uptake. A similar distribution of trace metal bioaccessibility in road dust from different functional areas is attributable to their similar concentrations of chemical species [14]. The typically higher bioaccessible fractions of trace metals in the gastric phase rather than the intestinal phase are explained by the much lower pH value (2.5) of the gastric phase, in which the adsorbed-associated and carbonate mineral-associated trace metals are readily dissolvable [56,57]. Consequently, the high bioaccessibility of Zn and Cd was a result of their dominant species being the carbonate-bound fraction [57]. The decreasing Cd and Pb in the intestinal stage may also be a result of their coprecipitation retention with dissolved Fe in the gastric phase [56]. The order of bioaccessible fractions in our study is similar to a previous study, i.e., Zn > Cd > Pb > Cu and Cr in the gastrointestinal extract of road dust from the Jamshedpur (industrial region) and Ranchi (commercial region), India [58]. It should be noted that the IVG bioaccessible As in road dust was much higher than the potential mobilizable fractions identified in the sequential extraction experiment, revealing that the sequential residual As may also be dissolved in the biological matrix [23]. Furthermore, Cd in road dust from agricultural fields showed the highest bioaccessible content, revealing a large health risk to local farmers.

When the IVG bioaccessible fraction was used in the calculation of health risk, the noncarcinogenic risk value for children largely decreased from 1.2 to 0.39 at a cumulative probability of 95%, showing no concern of noncarcinogenic risk. This comparison demonstrates that a bioaccessibility test is necessary before an accurate health risk of environmental samples [59]. Although road dust showed no significant enrichment of As and Cr, they showed the highest contribution to the noncarcinogenic health risk, mostly due to their high toxicity [13,47,58]. The highest carcinogenic risk was contributed by As, Cd, Cr, and Pb, consistent with the typical urban environment [13,58]. In comparison with the background value, the As in road dust was not severely contaminated and may originate from naturally weathering soil [10]. Road dust can be resuspended in the air and redeposited in surrounding soil or even indoor rooms [60], posing further risks to the human body. The exact sources of these four trace metals, particularly As, with high carcinogenic risk should be further investigated for appropriate management and control work.

## 5. Conclusions

Geochemical enrichment, chemical sequential species, IVG bioaccessibility, and the health risk assessment of trace metals in road dust from the megacity of Guangzhou, China, were systematically examined. Trace metals in road dust, including Zn, Cd, Cu, and Pb, highly exceeded the local background values and showed moderate to heavy contamination posing an ecological risk, probably due to traffic emissions and industrial activities in the central areas. Although most trace metals were associated with the Fe/Mn oxide-bound fraction, carbonate-bound fraction, and residual fraction, there was a large amount of Zn, Cd, Cu, and As dissolved in simulated gastrointestinal fluid. Accordingly, a cautionary carcinogenic risk was observed for the population based on the bioaccessibility-modified concentrations of trace metals, of which As, Cd, and Cr are the priority toxic elements.

## Figures and Tables

**Table 1:** Trace metal concentrations (mg/kg) in road dust from agricultural fields, industrial parks, and traffic intersections. The local background values were trace metal concentrations in soil obtained from a previous survey [45].

Sample (*n*)		As	Cd	Cr	Pb	Cu	Zn	Ni	V
Agricultural field (5)	Mean ± SD	19.2 ± 5.7	0.74 ± 0.74	55.7 ± 16.6	219 ± 314	46.9 ± 55.8	143 ± 57.5	15.1 ± 3.6	41.6 ± 6.4
	Median	22.8	0.43	50.5	147	139	460	32.0	44.5
	Range	12.8–23.8	0.31–2.05	35.2–73.5	77.4–259	45.9–171	236–558	24.9–46.0	34.2–47.3
Industrial park (23)	Mean ± SD	18.4 ± 12.6	0.74 ± 0.52	200 ± 266	166 ± 177	133 ± 154	640 ± 493	34.8 ± 33.0	37.3 ± 11.1
	Median	14	0.54	98.5	99.8	67.0	588	24.7	37.5
	Range	1.7–50.4	0.22–2.27	24.1–1260	29.9–806	16.2–625	46.9–1770	8.0–152	15.14–63.9
Traffic road (20)	Mean ± SD	25.1 ± 18.3	0.72 ± 0.72	116 ± 71.9	115 ± 90.9	116 ± 125	368 ± 166	41.2 ± 40.7	41.0 ± 13.1
	Median	21.7	0.48	106	79.6	86.1	332.2	31	40.0
	Range	4.7–61.2	0.23–3.14	34.4–347	47.1–440	23.8–599	162–761	10.7–201	18.3–61.8
Background		17.4	0.06	50.5	36.0	17.0	47.3	14.4	70.7

**Table 2:** Noncarcinogenic risks of trace metals in road dust using total and bioaccessible content.

Road Dust	Population	Mean	Standard Deviation	95%
Total content	Children	6.1 × 10^−1^	3.5 × 10^−1^	1.2
	Adult women	3.1 × 10^−1^	2.1 × 10^−1^	6.9 × 10^−1^
	Adult men	2.6 × 10^−1^	1.8 × 10^−1^	5.9 × 10^−1^
Bioaccessible content	Children	6.6 × 10^−2^	1.1 × 10^−1^	2.7 × 10^−1^
	Adult women	6.6 × 10^−2^	6.1 × 10^−2^	1.7 × 10^−1^
	Adult men	5.8 × 10^−2^	5.2 × 10^−2^	1.4 × 10^−1^

**Table 3:** Carcinogenic risks of trace metals in road dust using total and bioaccessible content. The acceptable threshold is 1.0 × 10^−6^.

Road Dust	Population	Mean	Standard Deviation	95%
Total content	Children	4.3 × 10^−5^	3.4 × 10^−5^	1.0 × 10^−4^
	Adult women	2.3 × 10^−5^	1.9 × 10^−5^	5.9 × 10^−5^
	Adult men	2.0 × 10^−5^	1.7 × 10^−5^	5.0 × 10^−5^
Bioaccessible content	Children	9.0 × 10^−6^	4.2 × 10^−6^	1.6 × 10^−5^
	Adult women	6.1 × 10^−6^	2.6 × 10^−6^	1.0 × 10^−6^
	Adult men	5.6 × 10^−6^	2.4 × 10^−6^	9.5 × 10^−6^

## Data Availability

The associated dataset of the study is available upon request to the corresponding author.

## Supplementary Materials

The following supporting information can be downloaded at: https://www.mdpi.com/article/10.3390/ijerph192315942/s1, Table S1. Pollution classification of the geoaccumulation index (I_geo_); Table S2. Improved Nemerow Index (INI) classification levels; Table S3. Parameters used in the health risk model. References [61,62,63] are cited in supplementary materials.
