# Supplementary material for: Geochemical Contamination, Speciation, and Bioaccessibility of Trace Metals in Road Dust of a Megacity (Guangzhou) in Southern China: Implications for Human Health
Source: Int J Environ Res Public Health. 2022 Nov 29;19(23):15942. doi: 10.3390/ijerph192315942 (PMC9736075; doi:10.3390/ijerph192315942)
Supplement: Supplementary file 1 [file ijerph-19-15942-s001.zip › ijerph-2042832-supplementary.pdf]

Supplementary Materials

Geochemical contamination, speciation, and bioaccessibility of trace metals in road dust of a megacity (Guangzhou) in southern China: Implications for human health

Fei Tang[1,2], Zhi Li[1], Yanping Zhao[3], Jia Sun[1], Jianteng Sun[2], Zhenghui Liu[2], Tangfu Xiao[1], Jinli Cui[1,2*]

[1]*Key Laboratory for Water Quality and Conservation of the Pearl River Delta, Ministry of Education; School of Environmental Science and Engineering, Guangzhou University, Guangzhou 510006, China*

[2]*Guangdong Provincial Key Laboratory of Petrochemical Pollution Processes and Control, School of Environmental Science and Engineering, Guangdong University of Petrochemical Technology, Maoming 525000, China*

[3]*Guangdong Provincial Key Laboratory of Chemical Measurement and Emergency Test Technology, Institute of Analysis, Guangdong Academy of Sciences (China National Analytical Center, Guangzhou), Guangzhou 510070, China*

Corresponding author (J.L. Cui): E-mail address: jlcui@gzhu.edu.cn; Tel.: +86-20-39366937. ORCID: 0000-0002-0299-7911

Table S1. Pollution classification of the geoaccumulation index (I_{geo}).

Contamination condition	Level	Value
Uncontaminated	Level 1	$I_{geo} \leq 0$
Uncontaminated to moderately pollution	Level 2	$0 < I_{geo} \leq 1$
Moderately pollution	Level 3	$1 < I_{geo} \leq 2$
Moderately to heavily contaminated	Level 4	$2 < I_{geo} \leq 3$
Heavily contaminated	Level 5	$3 < I_{geo} \leq 4$
Heavily to extremely contaminated	Level 6	$4 < I_{geo} \leq 5$
Extremely contaminated	Level 7	$I_{geo} > 5$

Table S2. Improved Nemerow Index (INI) classification levels.

Classification level	Contamination condition	Value
Class 0	Uncontaminated	INI < 0.5
Class 1	Uncontaminated to moderately contaminated	0.5 ≤ INI < 1
Class 2	Moderately contaminated	1 ≤ INI < 2
Class 3	Moderately to heavily contaminated	2 ≤ INI < 3
Class 4	Heavily contaminated	3 ≤ INI < 4

Class 5	Heavily to extremely contaminated	4 ≤ INI < 5
Class 6	Extremely contaminated	INI ≥ 5

Table S3. Parameters used in the health risk model.

Parameter	Definition	Unit	Value		Reference
			Adult	Children	
C	Concentration	mg/kg	-	-	-
IngR	Ingestion rate	mg/d	100	200	[1]
InhR	Inhalation rate	m^3/d	14.5	7．5	[1]
PEF	Particle emission factor	m^3/kg	1.36×109	1.36×109	[2]
AF	Adherence factor	$mg/m^2/day$	0.07	0.2	[2]
SA	Exposed skin area	cm^2	5700	2800	[3]
ABS	Dermal absorption factor		Non-cancer: 0.001<br>Cancer: 0.01		[3]
EF	Exposure frequency	day/year	350	350	[1]
ED	Exposure	year	25	6	[1]

	duration				
AT	Average time	days	Non-cancer: 26280		[1]
			Cancer: 9125		
BW	Body weight	kg	56.8	15.9	[1]
PEF	Particle emission factor	m^3/kg	$1.36×10^9$		[2]
ABS	Dermal absorption factor for Cd		0.001		[3]

References

1. MEP. *Technical Guidelines for Risk Assessment of Contaminated Sites Hj 25.3-2014*; Ministry of Environmental Protection of China: Beijing, China, 2014.
2. EPA. *Supplemental Guidance for Developing Soil Screening Levels for Superfund Sites*; Environmental Protection Agency, Washington, DC, USA, 2002.
3. USEPA. *Risk Assessment Guidance for Superfund Volume I: Human Health Evaluation Manual*; Part E, Supplemental Guidance for Dermal Risk Assessment); Office of Superfund Remediation and Technology Innovation: Washington, DC, USA, 2004.
